# Financial based agreements and performance based agreements: the Belgian experience

**DOI:** 10.1186/2052-3211-8-S1-O1

**Published:** 2015-10-05

**Authors:** Kim Pauwels, Isabelle Huys, Eveline Bielen, Sigrid Bormans, Robin Vincken, Henna Zheng, Minne Casteels, Steven Simoens

**Affiliations:** 1 Department of Pharmaceutical and Pharmacological Sciences, KU Leuven, Leuven, 3000, Belgium

## Background

In times of financial hardship, making innovative treatments affordable for society, accessible for patients and profitable for pharmaceutical companies is a challenge. Reimbursement is often threatened by uncertainties about the financial impact or evidence for a treatment. A contractual agreement between the payer and pharmaceutical company can link reimbursement to financial thresholds (financial-based agreements) or performance of a drug (performance-based agreements). The aim of this study was to investigate the experience with contractual agreements in Belgium.

## Methods

Qualitative research was performed to obtain insights into the perspectives of different stakeholders. Semi-structured interviews were conducted between September and December 2014. Interviewees were recruited through purposive sampling. Interviews were audio-recorded, verbatim transcribed and analyzed using the grounded theory approach.

## Results

Sixteen interviews were conducted, involving three representatives of the National Institute of Health and Disability Insurance (NIHDI), three representatives of sickness funds, seven representatives of a pharmaceutical company or pharmaceutical industry association, and three health care providers. All parties indicate that contractual agreements allow real-life data generation for treatments that would not be accessible for patients otherwise. The majority of agreements applied in Belgium establish financial compensations that a pharmaceutical company needs to accomplish in order to get a treatment reimbursed, with limited role for performance based aspects. Financial compensations remain confidential while the list price stays artificially high. The Belgian list prices influence other European prices due to the external reference pricing system. Industry representatives feel like financial based agreements are the only way to keep up the list price of the treatment. Representatives of the NIHDI notify the financial security that these agreements provide, while more data generation is allowed. In the light of financial based agreements, all stakeholders question the inherent meaning of the list price with regard to the value of the drug and the influence on external reference pricing. All parties emphasize that more attention for the added value of the treatment and a shift to more performance based agreements is required, although several hurdles still need to be conquered. Given current evolutions towards earlier market access, such as adaptive pathways and medical need programs, development of performance based schemes is a must.

## Conclusions

The European external reference price system drives the application of financial based agreements but at the same time, the value of the list price becomes meaningless. All stakeholders show eagerness for development and application of performance based agreements.

## Consent to publish

All participants of the study provided written consent to use the data for research purposes.

